# The Effects of Rhythm Training on Tennis Performance

**DOI:** 10.2478/v10078-012-0051-3

**Published:** 2012-07-04

**Authors:** Mustafa Söğüt, Sadettin Kirazci, Feza Korkusuz

**Affiliations:** 1 School of Physical Education and Sport, Kırıkkale University, Kırıkkale, TURKEY.; 2 Faculty of Education, Department of Physical Education and Sport, Middle East Technical University, Ankara, TURKEY.

**Keywords:** tennis, forehand consistency performance, rhythm training, rhythmic competence

## Abstract

Rhythm training is an integral part of sports. The purposes of the study were to analyze the effects of rhythm training on tennis performance and rhytmic competence of tennis players, to compare the improvement levels of tennis specific and general rhythm training and to examine the effects of shorter and longer tempos on rhythmic competence. Thirty university students whose mean score of International Tennis Number (ITN) was 7.3 (±0.9) were divided randomly into three sub-groups: Tennis Group, General Rhythm Training Group and Tennis-Specific Rhythm Training Group. The experimental procedure lasted 8 weeks. During this period, all groups had the same tennis training twice a week. The Tennis Group had regular tennis training sessions. In addition to regular tennis training sessions, the General Rhythm Training Group followed the general rhythm training sessions and the Tennis-Specific Rhythm Training Group had tennis-specific rhythm training. The measurement instruments were ITN, Rhythmic Competence Analysis Test and Untimed Consecutive Rally Test. The results indicated that participation in tennis-specific or general rhythm training resulted in progress in tennis playing levels, forehand consistency performance and rhythmic competence of the participants. On the other hand, attendance to the regular 8-week tennis training was enough to solely increase the tennis playing level but not sufficient to develop forehand consistency performance and rhythmic competence. Although the participants in the TRTG had better improvement scores than the ones in the GRTG, no significant difference was found between the rhythm training groups. The results also revealed that participants exhibited higher rhythmic competence scores on fast tempo compared to slow tempo.

## Introduction

Rhythm is the dynamic grouping, structuring and accentuation of sequential elements of a process, of which arrangement is determined by a required and/or personally selected temporal scheme (Schönborn, 2003). Previous studies reported the existence and importance of rhythm in sport skills. Weikart (1989) asserted that swimmers get their own beat by moving their arms and legs in a coordinated pattern of strokes and kicks. In addition, Zachopoulou et al. (2000) pointed out that swimming skills require performing a constant rhythm. Similarly, according to Laurence (2000), rhythmic abilities facilitate success in ballet. Moreover, dance movements are performed in a rhythmic structure and are affected by the elements of rhythm (Kirchner and Fishburne, 1995). Pica (1998) suggested that gymnastics, movement and rhythm are connected to each other. In addition, Borysiuk and Waskiewicz (2008) claimed that the fencers’ footwork rhythm provides information about the distance between the fighting opponents. Shaffer (1982) reported that sense of rhythm applied to ball games helps develop attitudes of calmness and fluency for performers. Zachopoulou et al. (2000) claimed that there is an external stimulus to which basketball and tennis players are forced to synchronize their movements and that production of rhythm for the same movements for a long duration is compulsory for athletes.

A growing body of literature also indicates the role and importance of rhythm in tennis. According to Bourquin (2003), the role of rhythm is important for tennis players to obtain harmonious movements. In addition, Segal (2005) claimed that, in professional tennis, good rhythm includes the capabilities of perfect control during impact, successful observation of ball movements, effortless transmission on the ball and effective timing. Furthermore, Zachopoulou et al. (2000) asserted that execution of motor skills in tennis requires synchronization of movements with an external stimulus, which is the ball trajectory. Likewise, Schönborn (2003) stated that rhythmic stroke production must be included for tennis training.

This study was triggered by the interesting findings of several experiments that have been conducted in the last decade. The first one (Zachopoulou et al., 2000) investigated the rhythmic ability of 50 tennis players (9.5 ± 5.2 years old), 53 basketball players (9.8 ± 6.3 years old), 52 swimmers (9.2 ± 4.2 years old), and 52 controls. Scores of children in the sport groups were found to be more accurate than the controls when rhythmic ability is considered, and the rhythmic ability test scores of tennis players were more accurate than those in the other three groups. It was claimed that all movements in tennis require distinct rhythmic structures, and practicing these movements creates opportunities for experiencing different tempos of rhythm, i.e. for rhythmic actions. The second study (Zachopoulou and Mantis, 2001) examined the effects of rhythm training on rhythmic ability and the forehand stability performance of tennis players. Fifty tennis players (23 girls and 27 boys at ages 8–10) participated in the study. The participants were subjected to a 10-week rhythm training program. The forehand groundstroke stability performance and the rhythmic ability of participants were measured before and after the training program. It was concluded that participation in the rhythm training caused improvement in rhythmic ability and the forehand stability performance of tennis players.

In this study, 30 university students, with a 7.3 (±0.9) mean score of International Tennis Number were divided randomly into three subgroups: Tennis Group (TG), General Rhythm Training Group (GRTG) and Tennis-Specific Rhythm Training Group (TRTG). The GRTG refers to training geared toward the development of fundamental locomotor and nonlocomotor skills aiming at synchronization of these skills with an external stimulus (metronome beats); whereas the TRTG means the rhythm training aiming at synchronization between movements that are specific to tennis and an external stimulus (metronome beats). The experimental procedure lasted 8 weeks. During this period, all groups had the same tennis training twice per week. The TG attended only regular tennis training sessions. In addition to regular tennis training sessions, the GRTG followed the general rhythm training sessions and the TRTG had tennis-specific rhythm training. The measurement instruments were ITN, Rhythmic Competence Analysis Test and Untimed Consecutive Rally Test.

Although previous studies emphasized the existence of rhythm, and thus the importance of rhythm training, in sport skills, there was no sufficient explanation or exercise prescription with regard to sport-specific rhythm training. This experimental study attempted to provide data in this field by testing the effects of rhythm training in tennis. In addition to its theoretical value, the present study also is expected to have practical significance; it attempts to achieve a new rhythm training approach by developing a new training system that can be used by coaches, physical education teachers and other tennis related staff. It was hypothesized that (1) participation in rhythm training (general or tennis-specific) would provide higher improvement scores on tennis performance and rhythmic competence than participation in regular tennis training, (2) participation in tennis-specific rhythm training would create higher improvement scores on tennis performance and rhythmic competence than participation in the general rhythm training, and (3) shorter and longer time intervals of tempo were expected to differentiate the rhythmic competence scores of the participants. The purpose of the study was to analyze the effects of rhythm training on tennis performance and rhytmic competence of tennis players, to compare the improvement levels of tennis specific and general rhythm training and to examine the effects of shorter and longer tempos on rhythmic competence.

## Methods

### Participants

Thirty university students (15 male and 15 female) whose tennis training histories varied between 2 and 42 months with a mean length of 12.8 months (±12.3), volunteered for this study. The mean age was of 23.1 (±2.3). Their mean International Tennis Number (ITN) was 7.3 (±0.9). Players at this level are fairly consistent while hitting medium paced shots, but are not yet comfortable with all strokes (Crespo et al., 2003). All participants were informed of the nature and purpose of the study both verbally and in a written form. All participants signed an informed consent form. Ethical permission was obtained from the Graduate School of Social Sciences of Middle East Technical University.

### Apparatus and task

ITN was used to determine the tennis playing level of the participants. Under this system, players are rated from ITN 1 to ITN 10. ITN 1 represents a high level play and ITN 10 indicates a player who is new to the game (Crespo et al., 2003). The participants performed a total of 42 strokes for the ITN test by applying basic tennis techniques.

The High/Scope Rhythmic Competence Analysis Test (RCAT) (Weikart, 1989) was used to assess the rhythmic competence of participants for both tempos of 50 and 100 beats per minute (bpm). Weikart (1989) designed RCAT in order to evaluate an individual’s rhythmic competence by testing his/her ability to perform a movement task to the underlying steady beat. A standart metronome was used for all tests and training procedures to arrange the tempos. RCAT was administered in a silent room. The participants were tested individually after they had been familiarized with the nature of the tasks and the testing environment. The scores were videotaped in order to analyze the performance in RCAT. Each participant was asked to synchronize a series of six movements for six times and a total of 36 movements were analyzed by the observers. The mean scores for each task were determined by averaging the scores of the two observers. The movements were as follows: 1) Patting the thighs with both hands at the same time, 2) Patting the thighs alternating the hands for each pat, 3) Walking the beat while still seated, 4) Walking the beat in place, 5) Walking forward, and 6) Walking backwards.

Two observers independently scored videotaped tests for each movement. They used a 1–3 scale where 3 was assigned to movements that have accurate synchronization, 2 to nearly synchronized movements, and 1 to nonsynchronized movements. In order to evaluate the intra-observer agreement, two observers analyzed the videotaped RCAT performances of 10 participants for each tempo. Furthermore, to assess the inter-observer agreement, the same observers analyzed videotaped RCAT performance of 10 participants twice at two different times. Observer agreement analysis for RCAT was calculated according to the following formula (Van Der Mars, 1989): Percentage of agreements = [Number of agreements / (Number of agreements + Disagreements)] × 100. Two observers agreed on 77 % intra-observer reliability for the tempo of 50 bpm and 79 % intra-observer reliability for the tempo of 100. Observers agreed on 75 % inter-observer reliability for the tempo of 50 bpm and 76 % for the tempo of 100 bpm.

The Untimed Consecutive Rally Test (UCRT) (Sherman, 1972) was used to analyze the forehand consistency performance of the participants. In Sherman’s study, the test reliability was .88, and the concurrent validity coefficient was .60. The testing area of UCRT was arranged with regard to instruction designed by Sherman (1972). A backboard or a smooth wall surface at least 3.03 m high and 6.09 m wide and a court or floor area extending outward from the board at least 9.14 m were required. A net line running parallel to the floor was located on a board 0.91 m above the floor area. A 2.13-m by 5.48-m target was placed on the board. It was 2.13 m above the net line. A parallel restraining line was located on the floor 6.40 m from the board.

In the UCRT, each participant was allowed one warm-up trial. All participants in each group were made to take their warm-up trial prior to the beginning of the first test trial. Each participant attempted to achieve the greatest number of consecutive rallies into the target in each trial. In starting the ball for the rally, the participant dropped the ball and hit it on the first bounce into the target area. All balls were to be contacted on or prior to the first bounce throughout the consecutive rally. Each participant had a total of three recorded trials. All participants, regardless of the group, were to finish the first trial before the second trial was taken. Failure to accomplish the following ended the consecutive rally: 1) To rally or volley the ball into the designated target area, 2) To contact the ball on the first bounce when starting the rally, 3) To contact the ball on the first bounce or prior to the bounce throughout the consecutive rally, and 4) To have at least one foot behind the restraining line. The score for each trial was the number of consecutive good rallies. The final score for the test was the mean of three trials. Two observers standing at different angles recorded the number of hits to the target area separately, keeping their own record sheets. Their record sheets were subsequently compared.

**Figure 1:**
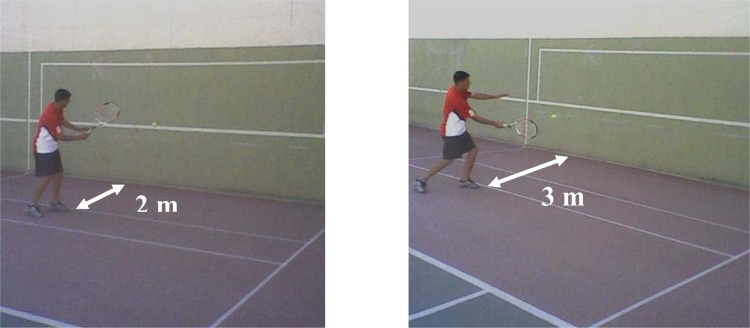
Forehand Consistency Tests a) 2 m b) 3 m (Photographs by Author)

Zachopoulou and Mantis (2001) applied the UCRT slightly modifying it in order to investigate the effects of rhythm training on the rhythmic competence and forehand consistency of tennis players. The researchers conducted UCRT in two distances (2 and 3 meters) from the training wall. This version of UCRT was preferred because of the specificity sought in the present study. As a matter of fact, restricting the players within a certain distance from the training wall makes the players adjust their movement to change in the ball’s trajectory and a change in its bouncing rhythm (Zachopoulou and Mantis, 2001). The test was thus applied at the distances of 2 m (Figure 1a) and 3 m (Figure 1 b) from the training wall.

### Procedure and design

The participants were tested on a one by one basis and all tests comprised of practice trials. Each participant was pre-tested on three tests in the following order: ITN, RCAT and UCRT. The participants were randomly assigned into one of the experimental groups provided that there was an equal number of males and females in each group: Tennis Group (TG), General Rhythm Training Group (GRTG), and Tennis-Specific Rhythm Training Group (TRTG). Each group consisted of 5 male and 5 female participants. The experimental procedure lasted 8 weeks. During this period, all groups performed the same tennis training program two times per week. The TG continued only regular tennis training sessions. The GRTG followed the general rhythm training sessions in addition to regular tennis training, two times per week for 15 minutes at the beginning of their training sessions. The TRTG had additional tennis-specific rhythm training two times per week for 15 minutes at the beginning of the regular tennis training sessions. For diagnostic purposes, a mid-test was applied at the end of the fourth week. The final measurements were performed after the 8-week training program was completed.

### Training procedures

All groups had the same tennis training program two times per week for 75 minutes. Each session started with a general and sport-specific warm-up and continued with a practice of basic tennis strokes. A pilot test was administered to determine the appropriate tempos of rhythmic movements for the rhythm trainings. It was observed that synchronization between rhythmic movements and the steady beat of the metronome became easier when the tempo was higher. Therefore, the range of tempo for rhythm trainings decreased gradually from faster to slower. Additionally, because of the nature of such exercises as bouncing the tennis ball with hand and racket, the tennis-specific rhythm training was performed with two different ranges of tempo: fast and slow. The slow tempos were between 45 and 55 bpm and the fast ones were between 80 and 120 bpm. These ranges of tempos were also set for the rhythmic movements for general rhythm training as parallel to tennis-specific rhythm training. All training sessions started with slow movements and continued with fast movements. Table 1 clarifies the gradual progression of tempos for rhythm trainings.

The programs of the GRTG and TRTG were designed taking into consideration the previous studies (Weikart, 1989; Zachopoulou and Mantis, 2001). The eight-week general rhythm training (Table 2) including several locomotor and nonlocomotor movements that were synchronized with the beats of the metronome was performed only by the GRTG. The general rhythm training was administered before the regular tennis training and conducted twice a week for fifteen minutes.

The tennis-specific rhythm training consisted of several tennis-specific movements (Table 3), which were synchronized with the beats of the metronome and was performed by the TRTG for eight weeks. The training was applied before tennis training and conducted twice a week for fifteen minutes.

### Statistical analysis

Parametric tests were initially supposed to be used in the analysis of the research data. However, non-parametric tests were conducted for the rest of the statistical process, since the Homogeneity of Variance assumption of One-Way ANOVA was violated. Thus, the Kruskal-Wallis Test was used to calculate the possible differences between initial scores and also to compare the improvement scores of groups. Afterwards, the Wilcoxon Test was used to examine the differences between initial, mid, and final test scores within each group. Finally, the Mann-Whitney U Test was conducted to determine the pairwise comparisons of groups for improvement scores and to analyze the rhythmic competence scores of participants for different tempos.

## Results

The Kruskal – Wallis Test results of groups for improvement scores were presented in Table 4. The results showed that there were significant differences among the UCRT (3m) and RCAT (50) scores of groups. Moreover, there were no significant differences among groups with respect to other parameters.

The Mann-Whitney U Test results indicated that there was a significant difference in slow tempo rhythm test scores (z=−2.15, *p*<.05) between the GRTG and the TG. Participants in the GRTG had higher improvement scores in the slow tempo rhythm test than the participants in the TG. There was no significant difference in the other parameters between groups. The results also demonstrated that there were significant differences in forehand consistency test (3m) scores (z=−2.79, *p*<.01) and slow tempo rhythm test scores (z=−3.51, *p*<.01) between the TRTG and the TG. The participants in the TRTG had higher improvement scores in the forehand consistency test (3m) and in the slow tempo rhythm test than the participants in the TG. The groups did not differ significantly in the other parameters. Although the participants in the TRTG had better improvement scores on forehand consistency, rhythmic competence, and ITN than the ones in the GRTG, no significant difference was found between rhythm training groups. The Mann-Whitney U Test results of the pre-test rhythmic competence scores of participants for the tempos 50 (bpm) and 100 (bpm) was z*=*−2.99, *p<*.01, which was significant. According to these results, synchronization of participants’ movements to the external stimulus was more precise at fast tempo than at slow tempo.

## Discussion

The results revealed that the participants in rhythm groups (GRTG and TRTG) improved their UCRT (2m) performance significantly after the training program. No significant difference was found between the pre-test and post-test results of the participants in the TG. Although no significant differences were found among groups, the participants in the TRTG improved their performance more than the participants in the TG at a very close significance level (*p* = .07). The duration of the training period might be a restrictive factor for improvement. If the participants had been exposed to rhythm training for more than eight weeks, there could have been significant differences between groups who had rhythm training and the TG. The results also showed that the participants in the TRTG improved their UCRT (3m) performance significantly more than the performance of the participants in the TG. According to Thaut (2005), practicing rhythmic activities not only regulates our movement but also provides opportunities to execute that movement more efficiently and precisely. Additionally, Zachopoulou et al. (2003) suggested that progression of the rhythmic ability leads to an improvement of motor coordination. It was reported that rhythm training also regulates the timing of the sequence of muscle contractions that produce movement (Thaut, 2005). Reid et al. (2003) asserted that during stroke production, it is very important for a tennis player to control the movements of different body segments and coordinate the contractions of different muscle groups. The tennis-specific rhythm training was a more effective way to enhance the forehand consistency performance than the general rhythm training. Throughout the training period, the participants in the TRTG performed nonlocomotor, locomotor and integrated rhythmic movements using their rackets, balls or both. On the other hand, the participants in the GRTG practiced with only nonlocmotor and locomotor rhythmic movements.

According to Zachopoulou and Mantis (2001), a change in distance forces players to adapt their movement to a change in the ball’s trajectory and a change in its bouncing rhythm. In this study, the two distances of the UCRT made a difference in the success of the participants. The results revealed that participants were better at the distance of 3 m than at the distance of 2 m. In other words, when the distance was longer, the participants had more time to synchronize their movement to the approaching ball, thus the movement was executed with greater accuracy.

The results indicated that the participants in the rhythm groups improved their RCAT (50 bpm) performance significantly more than the performance of the participants in the TG. In other words, participation in either the general or the sport-specific rhythmic activities yielded the development of rhythmic competence performance. The results of the study were in line with the findings of Wight (1937), Trump (1987), Weikart (1989), Zachopoulou and Mantis (2001), and Zachopoulou et al. (2003). They pointed out that the development of rhythmic ability is considerably related to training. According to Gallahue (1982), practicing with locomotor and nonlocomotor activities to different tempos, intensities, and accents provides an opportunity to enhance the fundamental elements of rhythm as well as skills in the movements. Although the participants in the TRTG had better scores than those in the GRTG, no significant difference was found between the rhythm groups.

The results showed that the participants in rhythm groups improved their RCAT (100 bpm) performance significantly after the training period. There was no significant difference between the pre-test and post-test results of the participants in the TG. Although the participants in rhythm groups had better scores than the participants in the TG, no significant difference was found between rhythm groups and the TG for the improvement scores. In other words, participation in the general or the sport-specific rhythm training could not bring about statistical improvement of rhythmic competence performance for the fast tempo. Additionally, there was no significant difference between the two rhythm groups. This finding might be explained with the similarity of time intervals between metronome beats for the fast tempo test (600 msec) and preferred tempo, which was determined by previous investigations as approximately 600 msec (Fraisse, 1982; Kumai and Sugai, 1997; Baruch et al., 2004). Baruch et al. (2004) determined a zone of preferred tempo in adults. A total of 60 male and 60 female participants at a mean age of 21 participated in the study. They concluded that the zone of preferred tempo for participants centered around 600 msec. In addition, Kumai (1999) found the same time interval for the participants with mental retardation. According to Smith (1999), biological processes like breathing, walking and heartbeat play a very important role in shaping the time duration for the preferred tempo. Because the participants responded to the fast tempo naturally, it was difficult to manipulate it through training. In contrast, at the slow tempo, they were asked to synchronize their movements with the exposed rhythm.

The results revealed that the mean ITN scores of each group increased significantly after the training program. Although the participants in the TRTG improved their performance more than the participants in the TG at a very close significance level (*p* = .05), no significant difference was found among groups. Moreover, there was no statistical difference between ITN scores of participants in the rhythm groups. Participation in regular tennis training enhanced the tennis playing level of all participants regardless of the groups.

The results also revealed that the synchronization of participants’ movement with the external stimulus was more precise at fast tempo than at slow tempo. The mean pre-test score for the slow tempo was 1.8 (±0.4) and 2.2 (±0.5) for the fast tempo. Because the interval of preferred tempo was in correspondence with the interval of the fast tempo test, the participants performed better at fast tempo than at slow tempo. This result is in accordance with the findings of previous investigations (Ellis, 1992; Zachopoulou et al., 2000; Mastrokalou and Hatziharitos, 2007).

## Conclusion

In conclusion, this is the first study that developed tennis specific rhythm training and assessed its effects on tennis performance. This experimental study focused on the importance of tennis-specific rhythm training. The effects of regular tennis training, general rhythm training and tennis-specific rhythm training on rhythm and tennis performance were investigated. At this point, the present study seems to be a leading study that developed a tennis specific rhythm training and assessed its effects on tennis performance. It can be concluded that tennis-specific rhythm training is a more effective method in order to enhance these parameters than general rhythm training. Since the generalization of the results of this study is limited to tennis, it is strongly recommended that future studies explore new approaches to sport-specific rhythm training

## Figures and Tables

**Table 1:** Training Progression Table for the Tempos of Rhythm Trainings

Slow Movements		Fast Movements	
Week	Tempo	Week	Tempo
1–2	55	1–2	120
3–5	50	3–5	100
6–8	45	6–8	80

**Table 2:** Rhythmic Movements for General Rhythm Training

**Slow Movements (45–55 bpm range)**	**Duration (minute)**

Side Jumping	1.5
Hand Clapping	2
Front and Back Jumping	1.5
Walking In Place	2

**Fast Movements (80–120 bpm range)**	**Duration (minute)**

Hand Clapping	2
Side Walking	2
Walking Forward and Backward	4

**Table 3:** Rhythmic Movements for Tennis-Specific Rhythm Training

**Slow Movements (45–55 bpm range)**	**Duration (minute)**

Bouncing tennis balls with both hands at the same time	1
Bouncing balls by alternating hands	1
Bouncing balls with both hands at the same time while walking	1
Bouncing balls by alternating hands while walking	1
Bouncing the ball with a racket using forehand and backhand groundstroke	2

**Fast Movements (80–120 bpm range)**	**Duration (minute)**

Performing forehand and backhand strokes, in four phases, without hitting the ball	3
Bouncing the ball with forehand and backhand volley synchronized with metronome beats	2
Performing ground strokes, without hitting the ball, with steps synchronized with metronome beats	4

**Table 4:** Kruskal – Wallis Results of Groups for Improvement Scores

Group/Tests	TG		GRTG		TRTG		χ^2^	*P*
	*M*	SD	*M*	SD	*M*	SD		
ITN	−1.0	1.0	−1.4	0.8	−1.9	0.7	3.98	0.136
UCRT 2m	12.4	42.7	23.2	27.2	37.0	29.6	3.12	0.210
UCRT 3m	12.8	20.5	43.0	56.0	57.6	39.0	7.24	0.027^*^
RCAT 50	0.1	0.3	0.7	0.6	0.9	0.3	11.73	0.003^**^
RCAT 100	0.3	0.5	0.5	0.3	0.6	0.6	1.62	0.443

* p < .05,

** p < .01
